# Crop/Plant Modeling Supports Plant Breeding: I. Optimization of Environmental Factors in Accelerating Crop Growth and Development for Speed Breeding

**DOI:** 10.34133/plantphenomics.0099

**Published:** 2023-10-09

**Authors:** Yi Yu, Qin Cheng, Fei Wang, Yulei Zhu, Xiaoguang Shang, Ashley Jones, Haohua He, Youhong Song

**Affiliations:** ^1^ Anhui Agricultural University, School of Agronomy, Hefei, Anhui Province 230036, China.; ^2^ Jiangxi Agricultural University, School of Agricultural Sciences, Nanchang, Jiangxi Province 330045, China.; ^3^ State Key Laboratory of Crop Genetics & Germplasm Enhancement and Utilization, Nanjing Agricultural University, Nanjing, Jiangsu Province 210095, China.; ^4^ The Australian National University, Research School of Biology, Canberra, ACT 2601, Australia.; ^5^ The University of Queensland, Queensland Alliance for Agriculture and Food Innovation, Centre for Crop Science, Brisbane, QLD, Australia.

## Abstract

The environmental conditions in customered speed breeding practice are, to some extent, empirical and, thus, can be further optimized. Crop and plant models have been developed as powerful tools in predicting growth and development under various environments for extensive crop species. To improve speed breeding, crop models can be used to predict the phenotypes resulted from genotype by environment by management at the population level, while plant models can be used to examine 3-dimensional plant architectural development by microenvironments at the organ level. By justifying the simulations via numerous virtual trials using models in testing genotype × environment × management, an optimized combination of environmental factors in achieving desired plant phenotypes can be quickly determined. Artificial intelligence in assisting for optimization is also discussed. We admit that the appropriate modifications on modeling algorithms or adding new modules may be necessary in optimizing speed breeding for specific uses. Overall, this review demonstrates that crop and plant models are promising tools in providing the optimized combinations of environment factors in advancing crop growth and development for speed breeding.

## Introduction

It is estimated that global population will reach 10 billion by 2050 [1]; meanwhile, a diet for animal-based products is overwhelmingly desired, which consumes more grains. Hence, global food demand is expected to double by 2050 [2,3]. Ever-changing environments caused by climate change are predicted to be occurring more frequently in future, which will seriously compromise crop production [4–7]. Agricultural resource constraints and climate change pose enormous challenges in achieving global food security. The development and adoption of ideal crop varieties with high yielding and strong biotic/abiotic resistance will be a key solution in addressing these challenges. Given the current pressure of worldwide food security, crop breeders and plant biologists have strived in identifying and utilizing traits or genes for the desired phenotypes in breeding crops but have been hindered by the duration of a crop generation.

Speed breeding (SB) is a technique of advancing crop generation mainly by regulating light and temperature in a controlled environment [8]. In the early 1980s, continuous light regime has been used to accelerate crop growth and development, by which, NASA has achieved rapid wheat life cycle in space and successfully developed a wheat variety of USU-Apogee with the traits of dwarf and high yield [9]. Recently, Hickey from the University of Queensland and colleagues first proposed and implemented the SB technology in a controlled environment, providing a way to produce crop generations in a short while [10]. Notably, SB has been successfully used in breeding processes for crop modification [11,12]. In addition, the crop generation number per year can be further increased by integrating selection methods including single seed descent, single pod descent, and single plant selection [8,13–15].

The transition from vegetative to reproductive growth in crops can be achieved by regulating environmental cues, including photoperiod, light quality, available water, and temperature, allowing their own developmental stages to synchronize with changes in light, temperature, and water resources [16]. Reasonable prolongation of photoperiod in SB accelerates phenological events and intensifies the physiological activities in crops [17,18]. However, environmental combinations including irradiance, photoperiod, and temperature, to some extent, may rely on individual specific experimental experiences, so there is room for the optimization in SB protocols. Accordingly, the model-assisted design in achieving optimal environmental conditions for crop growth and development will be particularly important. Crop and plant models are tools in predicting crop growth and development response to variable environments under open and controlled conditions [19]. Crop models are focused on simulating crop growth, development, and yield under various environments. While plant models are developed on the basis of crop models, with a greater emphasis on embracing 3-dimensional (3D) organ characteristics. Hence, both crop and plant modeling can supply various phenotypic dynamics. Thus, both models have been frequently used to run virtual experiments in testing the impact of given changes under specific conditions on crop growth, development, and 3D structural phenotypes [20–22]. Ideal environmental factor combination can be identified using crop models by adjusting and screening the input environmental factors based on the desired output crop phenotypic data [23–31]. Plant models precisely describe plant organogenesis, morphogenesis, and architectural development under various microenvironments at organ level, facilitating the investigation of the interaction among plant architecture at organ scale and microenvironments.

Conventional breeding transfers the excellent traits of both parents, including stress resistance and high yielding, to offspring through hybridization or backcrossing combined with strict phenotypic analysis, resulting in elite crop varieties [32]. This procedure has a long breeding cycle and involves a large amount of phenotyping work. The rise of molecular breeding techniques, including genome-wide association study [33], genome selection [34], and genotyping by sequencing [35], provides an excellent way for breeders to identify the association between genes and phenotypes. However, the development of parental lines based on these breeding techniques requires 4 to 6 crop generations, which becomes the bottleneck for the development of elite varieties [32]. Therefore, the application of SB technology enables the acceleration of stress-resistant or high-yielding-related gene identification by shortening the crop generation duration, thus achieving rapid development of ideal crop varieties by genetic engineering [36]. Wherein, the application of crop and plant models allows the identification of an ideal environmental factor combination for SB optimization. Hence, we argue that crop and plant models will be invaluable tools in optimizing the protocols in SB for customered research purposes. This review will illustrate the feasibility by describing how to use both models in optimizing the environmental conditions for SB.

## Overview of SB

**Fig. 1.**
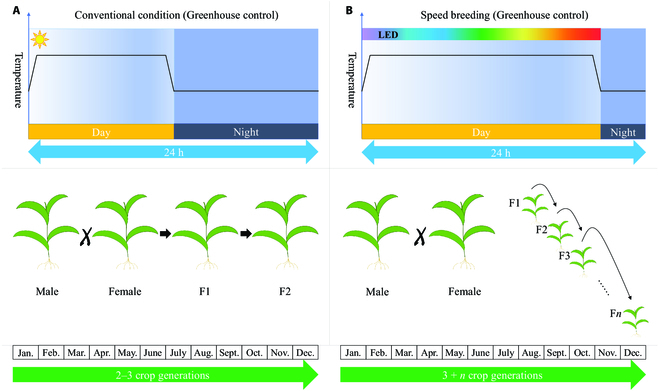
Diagram of SB accelerating the development of crop varieties. (A) Configuration of conventional conditions in the greenhouse, including given photoperiod and suitable crop temperatures from experiments, for achieving only 2 to 3 generations per year. (B) Configuration of optimized conditions in the greenhouse including suitable temperature, variable light period, intensity, spectrum, and CO_2_ replenishment by facilities, for achieving 3 + *n* crop generations per year.

Conventional breeding processes can take numerous generations to potentially produce a novel crop variety [37]. No doubt, the duration of every generation severely limits the development of new crop varieties [37,38]. SB reduces time of a crop generation by adjusting environmental factors such as photoperiod, light quality, light intensity, and temperature [8], which greatly relieves the constraint due to the lengthy crop generation time during the development of new varieties (see Fig. 1) [8,10]. A number of SB protocols have been developed for various crop species (see Table S1).

Breeding work based on SB has been successfully done in crops such as wheat [11], rice [12], and barley [13]. In wheat, by combining multitrait phenotyping techniques and SB, crop improvement in traits, such as root structure, resistance to crown rot, resistance to leaf rust, and plant height, can be achieved in a short time frame compared to conventional methods, by continuous screening for 4 generations every year [11]. A case study from rice showed that, the salt-tolerance *hst1* gene was rapidly introduced into the genetic background of a high-yielding rice variety by SB of 4 to 5 rice generations per year combined with single-nucleotide polymorphism-marker-assisted selection [12]. For barley, a variety with multiple disease resistance was bred within only 2 years using the strategy of backcross combined with multitrait phenotype screening based on SB [13]. It can be seen that crop development can be more effectively promoted by the combination of modern breeding technologies and SB.

## SB Appeals for Optimization Tools

Although the concept of SB is proposed, precision SB strategies should be developed for every specific crop type, which requires tedious examination of growth conditions. SB is carried out in greenhouse, in which the environment is rigorously controlled and depends on individual experimental experiences based on published literature. In the previous practice of SB, whether the applied environmental factor set is the optimal configuration remains to be determined [8,17].

Photoperiod is an important regulator for crop growth and development, controlling the transition between critical growth stages such as vegetative, flowering, maturity, and senescence [39]. Furthermore, the strength and duration of leaf photosynthesis in crops are also determined by photoperiod [40]. Therefore, photoperiod is a key factor determining SB performance. According to crop requirements for photoperiod, there are 3 types of crops, i.e., long day, day neutral, and short day. Light duration beyond crop critical day length promotes flowering in long-day crops, inhibits the flowering in short-day crops, but has little effect in day-neutral crops [41], while short-day treatment produces opposite consequences. It can be seen that for SB optimization, it is necessary to pursue an appropriate photoperiod critical point, minimizing crop generation time without causing growth disorders.

In addition to photoperiod, light intensity, properties (direct/diffuse light), quality, and direction affect SB performance by regulating physiological processes in crops. Excessive or insufficient light intensity restricts leaf photosynthesis in crops. For instance, when light intensity is oversaturated, the light capture in plant leaves exceeds the fixation of CO_2_ in dark photosynthesis, causing damage to crops due to active oxygens [42,43]. Direct light with a high intensity level often accompanies light resource concentration, resulting in the waste of light quantum, as well as less light saturation and radiation utilization efficiency (RUE) [44,45]. However, the diffuse light with same intensity level effectively avoids the constraint of light saturation and improves the RUE by evenly distributing light among all leaves in the canopy [46]. Hence, crops with narrow and deep canopies may have more advantages under direct light [47]. Light quality, the composition and proportion of light spectrum in the greenhouse, affects the performance of SB by regulating flowering time and morphogenesis [48]. Red light is the most effective for photosynthesis, which can stimulate bud growth and increase branches [49,50]. Blue light inhibits leaf expansion and stem elongation. The capacity of green light penetration within the canopy is strong, which can enhance the photosynthesis in leaves at the bottom of the canopy [51]. The stronger far-red light increases plant internode length, petiole length, and plant height, thus affecting canopy photosynthesis [52–55]. An optimized spectral proportion is required to maximize crop production. For example, when low-red/far-red light is imposed, plant shade avoidance response is triggered, and the development of leaves and storage organs is thus inhibited [56]. A recent study revealed that the flowering and maturation of short-day crops such as soybean and rice can be promoted by adjusting light quality. In comparison to 2 to 3 generations normally, growing soybean in 10-h photoperiods with blue-light enrichment and far-red-light depravation, enabled up to 5 generations to be bred each year [57]. The light direction may also play an important role in regulating crop growth and development during SB implementation. For example, crop phenotypes including leaf area, leaf number, stomatal characteristics, leaf angle, and plant height were reported to be affected by top, side, and bottom-up light [58–62].

Apart from light conditions, atmosphere composition and environmental temperature also have important roles in SB optimization. The light saturation point and photosynthetic capacity of crops, especially in C3 crops, can be effectively improved by supplementing CO_2_ in a controllable greenhouse, on the basis of moderately increased light intensity (direct/diffuse) [63]. In the context of sufficient light intensity and CO_2_, the optimum temperature for crop photosynthesis should be adjusted to a greater temperature [64]. Changes in light, temperature, and CO_2_ further affect crop transpiration, thus driving changes in water demand. The nitrogen fertilizer level is also an important factor in determining crop photosynthesis, by the facilitation of synthesis of enzymes and proteins [65–67].

It becomes clear that the development of precision SB strategies needs to optimize multiple environmental factors and their combinations. However, identifying these optimal combinations needs a huge investment of time, energy, and economy. If there is a predictive tool that can be used to effectively analyze the combination of environmental factors, the potential of SB can be realized in a more effective and economical way.

## The Ability of Crop and Plant Modeling in Simulating Genotype × Environment × Management

Virtual experiments run by crop, and plant models can be used as an effective means to predict crop growth and development in response to environmental variables and to obtain the optimal combinations of environmental conditions required by SB strategies for crop development (see Table S2). Crop and plant models are relatively complementary in roles and functions. Crop models are focused on predicting growth, development, and grain yield under environmental variables, aiding for the guidance of agronomic management. The key physiological processes, including phenological development, organogenesis, leaf and canopy photosynthesis, water and nutrient absorption, and biomass distribution, can be simulated by their own independent modules [68,69]. Consequently, crop models can be used to conduct analysis for the interaction of genotypes, environmental cues, and management strategies.

The advent of plant models, i.e., functional-structural plant model (FSPM), has opened up a new field for the fine simulation of crop growth and development resulted from the interweaving and cyclic interaction between plant structure and physiological functions at the organ level in response to environment and management. A thorough and precise understanding of the relationship between crop structure, functions, and abiotic factors can be achieved in guiding crop production [70–73]. In FSPM, plant body is composed of collective organs by leaves, nodes, internodes, and axillary buds on a certain topological organization [74]. In the light module of FSPM, the light interception and distribution within canopy are determined on the basis of a variety of methods. For example, Beer Lambert’s law is often used in many plant models, in which light interception exponentially decreases with the canopy depth [75–77]. Furthermore, the nested radiosity approach has been developed to calculate the exchange of light energy among plant organs [74,77]. Monte Carlo ray tracing is based on the absorption, reflection, and transmission paths of a given light source in the canopy, which depends on the optical properties of the plant material, to describe the light energy transmission in plant the canopy [77]. The advent of full-spectrum ray tracer and its integration into modeling has further enhanced the simulation of light reflection, absorption, and transmission on the leaves in canopy, as well as the simulation of crop growth and development under different spectral composition. Hence, continuous debugging of the parameters giving to the models enables an ideal environment combination suitable for crop production to be inferred [78–80], whereby the optimization of SB strategies can be conducted by improving and calibrating the existing crop and plant models.

## Models Able to Assist for SB

To accelerate crop generation through SB, precise optimization needs to be performed regarding photoperiod, temperature, radiant intensity, CO_2_ concentration, and water supply. The achievement of ideal crop phenotypes, including enhanced photosynthesis and early flowering, is the prerequisite for SB optimization. Hence, crop and plant models are able to assist for SB by identifying an optimized environmental factor combination for SB based on phenotypic changes. Crop physiological phenotypes can be optimized using crop modeling by quantifying physiological processes according to the given environments, allowing decision support for crop management in controllable environment [81]. On the basis of the predictions in crop models, Chauhan et al. [82] pointed out that for wheat varieties with low sensitivity to photoperiod, appropriate increase in soil moisture is beneficial for accelerating the flowering time in wheat due to the achievement of optimal daily heat accumulation. Both tomato growth model (TOMGRO) and the integration of TOMGRO and Vanthoor are shown to be capable in simulating crop growth and development in the greenhouse, by which the planting and management regimes in greenhouse can be improved [83,84].

**Fig. 2.**
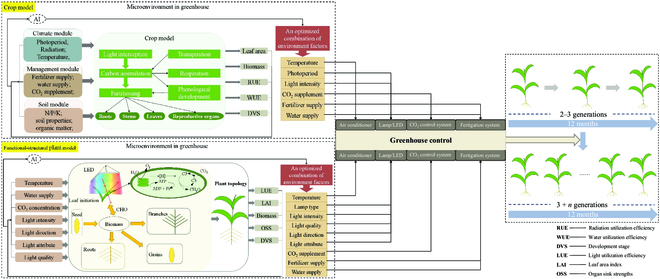
Schematic diagram of SB optimization by integrating crop and plant models. Crop growth and development driven by key physiological processes under environmental variables are simulated by independent functional modules; FSPM enables to display the plant physiology under environmental variables, ranging from cellular function to ecological function and ultimately reflecting in plant structure at the organ scale; SB can be optimized by debugging greenhouse equipment based on the optimized environmental conditions obtained through continuous adjustment or AI analysis of given environmental parameters from both models.

On the crop phenological development in SB, the phenological module in crop models allows the prediction of crop phenological developmental rate and stage using accumulated temperature along with photoperiod. Crop photosynthesis in SB can be described by the photosynthetic response curve in the carbon assimilation module of crop models, which takes temperature, leaf age, radiation intensity, day length, and CO_2_ concentration into account, where CO_2_ concentration and RUE regulate each other and jointly affect the crop transpiration efficiency and critical nitrogen concentration, by which biomass production and resource utilization under given conditions can be predicted [68]. Therefore, crop models enable to accelerate crop growth and development by the alteration and reset of given environmental parameters based on modeling feedbacks. The optimized environmental combination can be achieved by exercising different incremental combinations of day length, radiation intensity, temperature, CO_2_ concentration, and water supply. Hence, an ideal environmental factor set for optimizing SB can be obtained (see Fig. 2). Crop models have been proven to accurately predict the variation in crop growth and development due to changes in environmental factors such as radiation [85], fertilizer level [86], irrigation level [87,88], temperature [89], and CO_2_ concentration. For example, in the study of Mohanty et al. [90], the simulation results of Agricultural Production Systems sIMulator (APSIM) model showed that wheat grain yield would decrease by 8% for each degree of increase in the benchmark temperature, while wheat grain yield would increase by 33% upon CO_2_ increase by 500 parts per million. Furthermore, crop models have long been used by researchers to assist in crop breeding. For instance, Hammer et al. [91] used a maize crop model, i.e., APSIM-Maize, and found that root structure and water capture, rather than canopy structure, have a direct impact on maize biomass accumulation, which can be used for crop improvement in drought tolerance; APSIM sorghum model was used to evaluate genotype × environment × management effect on drought adaptation, indicating that maximizing the capture of production water and optimizing its distribution before and after anthesis are the key principles in designing sorghum varieties suitable for its cultivation in the northeastern region of Australia, predicting and identifying the fertile tiller number and maturity as the main direction for variety improvement [92].

Light quantity and quality are the decisive factors for crop biomass production through leaf photosynthesis, especially in totally enclosed greenhouses. With the aid of 3D ray-tracing light model and the biochemical model for C3 photosynthetic CO_2_ assimilation published by Farquhar, von Caemmerer, and Berry (FvCB), FSPM was successfully applied to guide the type, density, and location of the light in greenhouse for obtaining the ideal lighting strategy suitable for tomato growth [78]. The prediction of the impact of light properties (direct/diffuse) on canopy light interception was also completed in FSPM with radiosity and Monte Carlo ray-tracing-based light simulation algorithms [93]. Furthermore, FSPM has the ability to simulate crop growth and development in response to light spectral composition after the development of full-spectrum ray tracer and its integration into 3D modeling platform GroIMP [78]. In FSPM, the value of reflection, transmission and absorption for each spectrum on the leaves, the number of micromoles assimilated CO_2_ per micromole absorbed light, and the response parameters of plant structure to different spectral compositions are set in advance. Through this way, FSPM allows previewing the effects of light positions, directions, intensities, and properties on crop growth and development. For example, this study in investigating the impact of diffuse light proportion on cucumber growth via the simulation of 3D structural model showed that an increase of 226.1% in diffuse light proportion gave rise to an 34.4% increase in light interception; however, the diffusion caused a 56.8% reduction in radiation reaching the leaves, which reduced the advantage of diffuse light [94]. In addition, the study of Hitz et al. [95] revealed that lower blue-light intensity could increase soybean leaf area and biomass, which is favorable for rapid soybean production; interestingly, the use of light-emitting diodes with the low blue light of 60 μmol·m^−2^·s^−1^ contributed to a reduced energy consumption by 7% compared to the control. Hence, a reasonable lighting scheme for SB strategies can be made available by achieving a satisfactory output (see Fig. 2).Taken another example in the research by Kalaitzoglou et al. [96], 3D structural plant model was used to simulated the effects of far-red light with 4 phytochrome stationary state on tomato plant growth, indicating that continuous far-red light with 0.8 phytochrome stationary state supply can raise fruit yield due to increased leaf source intensity, which could increase total leaf area and accelerate flowering time. Although light parameters for different crops in the current FSPM, especially the spectrum, need to be further tested and calibrated, optimization support of light environments for SB can be provided by FSPM to some extent. It can be seen that the simulating accuracy with FSPM in crop growth and development based on the spectral composition of light should be further improved after continuous optimization and calibration. Interestingly, FSPM has been also used to guide breeding, such as designing ideal types that are beneficial for tomato light absorption and photosynthesis. With the leaf area index remaining unchanged, when the ratio between leaf length and leaf width changes from the default value of 1.02 to 1.5 times, it is predicted to be the ideal phenotype for tomatoes, with a light absorption value increasing by 8% in winter and 10% in summer [74].

To guide greenhouse equipment management with desired environmental conditions, a series of greenhouse models involving microclimate, lighting system, heating demand, and balancing the relationship between crop production and energy consumption in the greenhouse have been developed [97–99]. The integration of design elements in the greenhouse models, including greenhouse structure type, coverage type, outdoor shade screen, whitewash, thermal screen, heating system, cooling system, and CO_2_ enrichment system, allows for describing the impact of outdoor climate and greenhouse design on the microclimate inside the greenhouse [100]. Hence, the application of greenhouse models is of great value to the setting of greenhouse equipment during the practice of SB, thereby maintaining an ideal and stable environment inside the greenhouse. To optimize SB strategies, the setup of greenhouse equipment during the implementation of SB can be formulated by predicting the impact of outdoor climate conditions on the temperature, humidity, and CO_2_ in the greenhouse. A greenhouse model of GreenLight was also developed to evaluate the impact of radiant heat generated by the supplementary lighting of high-pressure sodium and light-emitting diode on the temperature in greenhouse [101]. By which, the temperature deviates between real-time temperature in greenhouse and ideal temperature for SB caused by external environment, different greenhouse types, and lamp types, and quantities can be predicted, assisting for proposing heat strategies.

## Future Prospects and Challenges in Integrating Crop and Plant Modeling for SB

Optimizing SB with the existing models must continuously adjust the environmental parameter datasets to infer an optimal combination of environmental factors by examining the simulations repeatedly. Artificial intelligence (AI) incorporated with computer science, mathematics, and engineering enables fast data analysis and provides desired outputs through continuous trainings and learnings [102,103]. Therefore, the integration of AI into crop and plant models improves the operation intelligence and predictive accuracy with models by enhancing the efficiency of processing and analysis in terms of complex input and output datasets. For example, to solve the complex nonlinear relationship between inputs and outputs within models, the both AI technologies, machine learning and deep learning, were used to provide interpretation for soybean trait prediction and assist feature selection [104]. Hence, the integration of AI is expected to assist crop and plant models in optimizing SB by improving the intelligence and simulation accuracy of modeling studies.

Despite crop and plant models having potential to optimize SB, there are some limitations in using the existing models. First of all, several core crop models including APSIM and Decision Support Systems for Agrotechnology Transfer (DSSAT) are developed on the basis of open environments, which are rarely reproduced in the greenhouse. In addition, most crop models available in the greenhouse are developed or applied for vegetable plants. Therefore, crop models need to be calibrated or modified to be used in the greenhouse. Next, there are few studies on applying crop and plant models to predict the effect of spectral composition on crop growth and development, especially for phenological development, notably crop flowering time. Therefore, the spectral parameters need to be incorporated into the further model development for SB. Finally, there is little research on the combined application of crop and plant models, although both are complementary in functions. Crop models and plant models for SB optimization need to be calibrated to have compatibility. Therefore, the original algorithms and program of crop and plant models need to be further modified or calibrated for the optimization of crop growth and development in SB.

## Conclusions and Future Insights

The application of SB to accelerate the development of new crop varieties with ideal traits has high potential to enhance future food security. SB requires accelerated plant growth and development by having an optimal combination of environmental factors, largely varying with crop species, and specific experimental experiences. The optimal management strategies in the greenhouse including water supply, nitrogen application, temperature, and CO_2_ concentration control can be accustomed for SB by crop models, while the light environment in the greenhouse including light quality, light intensity, the ratio of direct and diffuse light, and light direction can be optimized by plant models for SB. Notably, crop and plant models are promising tools in optimizing SB by (a) calibration and adaptation of model parameters to SB condition under greenhouse context, (b) use of ray tracer to improve the sensitivity to light quality and path, and (c) optimization of model algorithm through AI technology to improve predictive accuracy and operational intelligence. Hence, the collective use of both models facilitates the optimizations required to develop precision SB strategies. Overall, we propose that crop and plant models in system simulations provide a highly promising approach to optimizing SB, which enables the development of elite crop varieties needed for future food security.

## Supplementary Materials

Supplementary 1 Tables S1 and S2 References [105–143]
