# Supplementary material for: Crop/Plant Modeling Supports Plant Breeding: I. Optimization of Environmental Factors in Accelerating Crop Growth and Development for Speed Breeding
Source: Plant Phenomics. 2023 Oct 9;5:0099. doi: 10.34133/plantphenomics.0099 (PMC10561689; doi:10.34133/plantphenomics.0099)
Supplement: Supplementary 1 — Tables S1 and S2 References [105–143] [file plantphenomics.0099.f1.docx]

Table S1. List of speed breeding (SB) conditions and life cycle generations achieved for key plants of agricultural importance.

| Crop type | Temperature (day/night) | | SB conditions | Achieved generations | Reference |
| --- | --- | --- | --- | --- | --- |
| Wheat | 22/17 ^o^C | Extended photoperiod (22 h light/2 h dark) with single seed descent (SSD) | | 6 | Watson et al., 2018 [107] |
| Barley | 22/17 ^o^C | Extended photoperiod (22 h light/2 h dark) with SSD | | 6 | Watson et al., 2018 [107] |
| Rice | 30/25 ^o^C | A longer day-length (14/10 h light/dark) for first 30 days followed by a shorter day-length (10/14 h light/dark) to induce reproduction, tiller removal, and embryo rescue | | 4-5 | Rana et al., 2019 [12] |
| Pea | 22/17 ^o^C | Extended photoperiod (22 h light/2 h dark) with SSD | | 6 | Watson et al., 2018 [107] |
| Chickpea | 22/17 ^o^C | Extended photoperiod (22 h light/2 h dark) with SSD | | 6 | Watson et al., 2018 [107] |
| Lentil | 24/18 ^o^C | Extended photoperiod (20 h light/4 h dark) with SSD | | 5-6 | Lulsdorf and Banniza, 2018 [8] |
| Soybean | 28/28 ^o^C | 10 h photoperiod with a blue-light enriched, far-red-deprived | | 5 | Jaehne et al., 2020 [108] |
| Faba bean | 20/18 ^o^C | Early flowering by plant growth regulators | | 7 | Mobini et al., 2015 [109] |
| Lupin | 24/20 ^o^C | 20 h photoperiod accompanied by different light spectra (blue and far red-enriched LED lights and metal halide) | | 5 | Croser et al., 2016 [110] |
| Peanut | 32/22 ^o^C | Continuous (24 h) light conditions | | 4 | O'Connor et al., 2013 [111] |
| Canola | 22/17 ^o^C | Extended photoperiod (22 h light/2 h dark) with SSD | | 4 | Watson et al., 2018 [107] |
| Amaranth | / | A longer day-length (16 h, 35°C) conditions for strong vegetative growth followed by short day conditions (8 h, 30°C) for inducing early flowering | | 6 | Stetter et al., 2016 [112] |

Table S2. Currently available crop models and the parameters that are included (or excluded) from the model. N/A = not applicable.

|  | Crop model | | | | | | | | | | |
| --- | --- | --- | --- | --- | --- | --- | --- | --- | --- | --- | --- |
| Parameter | | APSIM | Reference | DSSAT | Reference | STICS | Reference | AquaCrop | Reference | WOFOST | Reference |
| Radiation | | ✓ | Dilla et al. 2018 [86] | ✓ | Xia et al., 2014 [113] | ✓ | Affholder et al., 2003 [114] | N/A | N/A | ✓ | Wang, Liu, and Shi, 2023 [115] |
| Temperature | | ✓ | Wang et al. 2018 [116] | ✓ | Xia et al., 2014 [113] | ✓ | Monestiez et al., 2001 [117] | ✓ | Ahmadi, Ghorra, and Sepaskhah, 2022 [118] | ✓ | Wang, Liu, and Shi, 2023 [115] |
| Rainfall and irrigation | | ✓ | Sun et al. 2019 [119] | ✓ | Xia et al., 2014 [113] | ✓ | Affholder et al., 2003 [114] | ✓ | Tavakoli, Moghadam, and Sepaskhah, 2015 [120] | ✓ | Wang, Liu, and Shi, 2023 [115] |
| Photoperiod | | ✓ | He et al., 2017 [121] | ✓ | Akinseye et al., 2017 [122] | ✓ | Folliard et al., 2004 [123] | N/A | N/A | ✓ | de Wit et al., 2019 [124] |
| CO_2_ concentration | | ✓ | Zhang et al., 2023 [125] | ✓ | Xia et al., 2014 [113] | ✓ | Li et al., 2011 [126] | ✓ | Kourat et al., 2021 [127] | ✓ | Wang, Liu, and Shi, 2023 [115] |
| Soil properties | | ✓ | Connolly et al., 2002 [128] | ✓ | Fry et al., 2017 [129] | ✓ | Affholder et al., 2003 [114] | ✓ | Kalumba et al., 2021 [130] | ✓ | de Wit et al., 2019 [124] |
| Soil fertility and fertilization | | ✓ | MacCarthy et al., 2015 [131] | ✓ | Aluoch et al., 2022 [132] | ✓ | Singh et al., 2014 [133] | ✓ | Rahimikhoob, Sohrabi, and Delshad, 2021 [134] | ✓ | Sun et al., 2023 [135] |
| Soil moisture content | | ✓ | Sun et al., 2019 [119] | ✓ | Xia et al., 2014 [113] | ✓ | Affholder et al., 2003 [114] | ✓ | Boudhina et al., 2019 [136] | ✓ | Dewenam et al., 2021 [137] |
| Soil evaporation | | N/A | N/A | N/A | N/A | N/A | N/A | ✓ | Boudhina et al., 2019 [136] | N/A | N/A |
| Soil salinity | | ✓ | Sarkar et al., 2022 [138] | ✓ | Liu, Mishra, and Yu, 2019 [139] | N/A | N/A | ✓ | Li et al., 2022 [140] | ✓ | Kroes and Supit, 2011 [141] |
| Crop evapotranspiration | | ✓ | Saddique et al., 2020 [142] | ✓ | Shelia et al., 2018 [143] | ✓ | Crepeau et al., 2021 [144] | ✓ | Boudhina et al., 2019 [136] | ✓ | Govindarajan, Ambujam, and Karunakaran, 2008 [145] |
| Cultivation strategy | | ✓ | Xu et al., 2021 [146] | ✓ | Nafi et al., 2021 [147] | ✓ | Affholder et al., 2003 [114] | ✓ | Xie et al., 2023 [148] | ✓ | Dewenam et al., 2021 [137] |
